# Increased risk of cutaneous lupus erythematosus flare during the late menopausal transition: A retrospective study of 64 women

**DOI:** 10.1016/j.jdin.2026.05.022

**Published:** 2026-06-08

**Authors:** Quentin Allibert, Alexandre Teboul, Gabrielle Sonigo, Antoine Louchez, Annick Barbaud, Jessica Fareau, Jean-David Bouaziz, Hélène Collinot, François Chasset

**Affiliations:** a Service de Dermatologie et Allergologie, Sorbonne Université, Faculté de médecine, AP-HP, Hôpital Tenon, Paris, France; b Dermatology, Saint-Louis Hospital, APHP and Paris Cité University, Paris, France; c Department of Gynecology and Obstetrics, AP-HP, GHU-Sud, Hospital Bicêtre, Le Kremlin Bicêtre, France; d Institut Cochin, Inserm U1016-CNRS UMR8104 - Université Paris Cité, Paris, France; e CIMI, INSERM U1135, Paris, France

**Keywords:** cutaneous lupus erythematosus, menopause, perimenopause

*To the Editor:* Sex hormones are considered to play a major role in lupus pathogenesis.^1^ Although some data suggest that systemic lupus erythematosus (SLE) is associated with decreased disease activity and reduced magnitude of flares after menopause,^1^ data on cutaneous lupus erythematosus (CLE) remain scarce.^2^ Compared with SLE, CLE is characterized by a lower female-to-male ratio (3-4/1 vs 8-9/1) and a higher age at onset.^3^ The objectives of this study were (1) to assess whether the menopausal transition impacts CLE activity and (2) whether CLE activity decreases after menopause. In a cohort of consecutive CLE patients seen between January 2021 and July 2023, women aged ≥45 years were included. Men and women aged <45 years were excluded. Participants completed a standardized questionnaire about the date of the final menstrual period (FMP), age at menstrual irregularity, climacteric symptoms, and use of menopausal hormone therapy. CLE subtypes, associated SLE, and CLE treatments were extracted from electronic medical records. Follow-up was divided into predefined 1-year windows relative to the FMP: perimenopause (Year −2 [Y−2] and Year −1 [Y−1]) and early postmenopause (Year +1 [Y+1]).^4^ In the absence of systematic longitudinal activity scores for all patients, treatment intensification was used as a pragmatic proxy for CLE flares. The primary analysis used mixed-effects logistic regression with a random intercept for patients (GLMM, binomial logit) to account for repeated measures and unbalanced follow-up. Results are reported as adjusted probabilities and odds ratios with 95% confidence intervals, using the reference year at age 40 as the comparator. Among 263 CLE patients, 64 women were included. Median age at CLE onset was 35 years (IQR, 29-43), and median age at last follow-up was 55 years (IQR, 50-62). Most patients were diagnosed with CLE before menopause (58/64, 91%). At last follow-up, 49 patients (77%) were postmenopausal, with a median age at menopause of 50 years (IQR, 45-51), and climacteric symptoms were reported by 40 of them (82%). Chronic CLE was the most frequent subtype (90%), and 48% fulfilled SLE classification criteria (Table I). Treatment intensification was significantly more frequent at Y−1 (OR, 3.07; 95% CI, 1.11-8.47; Supplementary Table I, available via Mendeley at https://data.mendeley.com/datasets/m6s45rpwys/1), with 45% intensification compared with 24% at reference (Fig 1). An exploratory treatment-change score model showed a similar trend (Supplementary Fig 2, available via Mendeley at https://data.mendeley.com/datasets/m6s45rpwys/1). Moreover, no significant reduction in treatment intensification was observed at last follow-up compared with the reference period (OR, 1.71; 95% CI, 0.67-4.40). Overall, we found a significant increase in CLE flares during late perimenopause. This finding is consistent with a previous study lacking longitudinal analysis.^2^ It has been suggested that hormonal imbalance preceding menopause may account for these flares. Moreover, the absence of a decreased flare rate at last follow-up suggests that CLE activity persists after menopause. This aligns with the LUMINA cohort, showing that menopause in SLE is associated only with a reduction in renal flares.^5^ Limitations include the retrospective design, potential recall bias, and the absence of longitudinal CLASI-A scoring and sex hormone measurements. Further prospective studies are needed, particularly to assess long-term trends across menopause.

**Table I:** Characteristics of the study population (overall cohort, *N* = 64)

Features	Overall population *N* = (64)
Age at CLE onset, y, median (IQR)	35 [29-43]
Age at last follow-up, y, median (IQR)	55 [50-62]
Fitzpatrick phototype	
I-IV, *n* (%)	47 (73)
V-VI, *n* (%)	17 (27)
CLE subtypes	
Acute, *n* (%)	3 (5)
Subacute, *n* (%)	12 (19)
Chronic, *n* (%)	58 (90)
Discoid, *n* (%)	51 (80)
Tumidus, *n* (%)	15 (23)
Chilblain, *n* (%)	5 (8)
Lupus panniculitis, *n* (%)	5 (8)
Several subtypes, *n* (%)	27 (42)
CLE activity at diagnosis	
CLASI-A <10, *n* (%)	46 (72)
CLASI-A ≥10, *n* (%)	18 (28)
Associated SLE∗	31 (48)
CLE treatment	
Number of treatment lines ever received, median (IQR)	3 [2-5]
Number of treatments at FMP, median (IQR)	1 [1-2]
Number of treatments at LFU, median (IQR)	1 [1-2]
Systemic treatments ever used, *n* (%)	
Hydroxychloroquine	63 (98)
Systemic corticosteroids	27 (42)
Methotrexate	29 (45)
Thalidomide/Lenalidomide	25 (39)
Belimumab	10 (16)
Anifrolumab	11 (17)
Mycophenolate mofetil	7 (11)
Other treatments†	19 (30)
Menopausal status at CLE diagnosis	
Premenopausal, *n* (%)	58 (91)
Postmenopausal, *n* (%)	6 (9)
Menopausal status at LFU	
No menopause, *n* (%)	15 (23)
Menopause, *n* (%)	49 (77)
Among menopausal patients	*N* = 49
Age at menopause, median (IQR)	50 [45-51]
Age menstrual irregularity, median (IQR)	47.5 [44-50]
Climacteric symptoms, *n* (%)	40 (82)
Active smoking at menopause, *n* (%)	23 (47)
Use of Menopausal Hormone Therapy, *n* (%)	6 (12)

*ACR*, American College of Rheumatology; *CLASI*, Cutaneous LE Disease Area and Severity Index; *CLE*, cutaneous lupus erythematosus; *EULAR*, European Alliance of Associations for Rheumatology; *SLE*, systemic lupus erythematosus.

∗ Based on 2019 EULAR/ACR Classification Criteria for Systemic Lupus Erythematosus.

† Dapsone, deucravacitinib, upadacitinib, litifilimab, rituximab, cyclophosphamide, azathioprine, apremilast, alitretinoin, isotretinoin.

**Fig 1:**
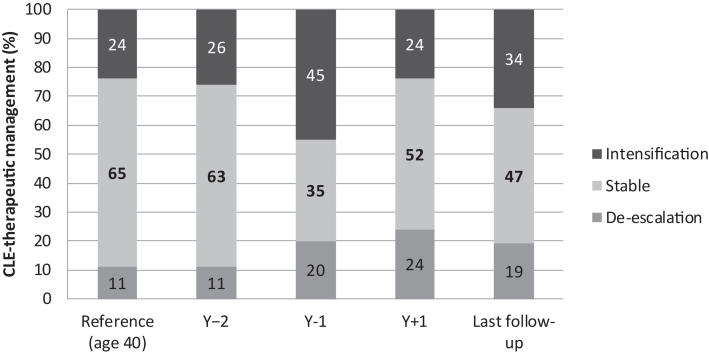
Changes in CLE therapeutic management across predefined 1-year windows relative to the final menstrual period (FMP). Bars represent the proportion of patients with treatment intensification, stability, or de-escalation during the reference year at age 40, Year −2 (Y−2), Year −1 (Y−1), Year +1 (Y+1; early postmenopause), and the last year of follow-up. Patients with ≥1 treatment intensification within a given 1-year window were classified as intensification. Treatment intensification was defined as at least 1 dose increase (eg, antimalarials such as hydroxychloroquine from 400 mg/day to 600 mg/day) or the addition or switch to a second- or third-line therapy (immunosuppressive agents, thalidomide/lenalidomide, or biologic therapies) within each 1-year window. The prescription of topical treatments was not considered as treatment intensification. De-escalation was defined as a decrease in antimalarial dose (eg, from 400 mg/day to 200 mg/day) or discontinuation of any second- or third-line therapy.

## Conflicts of interest

François Chasset has received grant/research support from AstraZeneca, BMS and GSK; participated in an advisory board related to lupus for AstraZeneca, GSK, Celgene, Merck, horizon therapeutics, Novartis, Kyowa Kirin and Principabio and received speaking fees and honoraria from AstraZeneca and GSK BMS related to lupus.
